# Latent classes of substance use precursors predict positive alcohol and cannabis expectancies one year later in Black and Latino early adolescents in the ABCD study

**DOI:** 10.1016/j.addbeh.2026.108702

**Published:** 2026-04-05

**Authors:** Tammy Chung, Nicole Kennelly, Shawn J. Latendresse, Margret Z. Powell, Carolyn E. Sartor

**Affiliations:** a Institute for Health, Health Care Policy and Aging Research; Rutgers Addiction Research Center; Rutgers University, New Brunswick, NJ, USA; b Baylor University, Waco, TX, USA

**Keywords:** Latent classes, Alcohol, Cannabis, Black, Latino, Adolescent

## Abstract

**Introduction:**

Precursors of alcohol and cannabis use, such as friend (dis)approval, have rarely been examined in person-centered analyses that can inform tailored prevention, particularly among Black and Latino youth. This study aimed to identify person-centered patterns (classes) of precursors of alcohol and cannabis use in Black and Latino youth, examine correlates across classes, and determine whether the classes predicted positive anticipated effects (expectancies) of alcohol and cannabis one year later.

**Methods:**

Latent class analyses used data from Black and Latino pre-adolescents in the Adolescent Brain Cognitive Development Study Follow-up Year 2 (n = 3,003; 31.0% Black, 69.0% Latino; mean age: 12.0, SE = 0.0). The 3-step method examined correlates of the classes (e.g., parental monitoring, cultural values) and used class membership to predict positive expectancies at Follow-up Year 3.

**Results:**

Three latent classes emerged: low risk (High friend disapproval/ moderate-high perceived risk of harm: 46.7%), high risk (Low friend disapproval/low perceived risk of harm: 10.5%), and a class characterized by protective (high friend disapproval) and risk-elevating (low perceived risk of harm) precursors (divergent class; 42.8%). The high (vs low) risk class reported greater externalizing behavior. The low risk (vs divergent) class was higher on an indicator of familism. Low risk (vs divergent) class membership predicted lower positive expectancies across substances, but low (vs high) risk class membership did not significantly predict expectancies.

**Conclusion:**

Findings underscore heterogeneity in the co-occurrence of precursors to alcohol and cannabis use among pre-adolescent Black and Latino youth and the relevance of distinct patterns of precursors in predicting positive expectancies.

## 1. Introduction

Prior to substance use onset, precursors of use such as perceived ease of access, friends’ disapproval of use, and risk of harms from use can signal a youth’s readiness to engage in alcohol and cannabis use (Nawi et al., 2021). Research on these precursors in adolescence, when initiation of substance use escalates, has examined commonly used substances, such as alcohol and cannabis, separately (Ahuja et al., 2022; Boyas et al., 2019). However, work examining alcohol and cannabis simultaneously identified differences in risk that were linked with race/ethnicity (Sartor et al., 2022). Important next steps for tailoring prevention involve identifying person-centered risk profiles across alcohol and cannabis, when considered together in understudied groups such as Black and Latino youth (Ayón et al., 2020).

In a risk factors framework (Nawi et al., 2021), key predictors of early use include perceived ease of access to substances (Miller et al., 2023), risk of harm due to use (Merianos et al., 2017), and friends’ disapproval of use (Johnson et al., 2023). Notably, differences by race/ethnicity have been found for some risk factors (Lee et al., 2021). For example, Black, relative to White, youth reported lower access to alcohol and cannabis (Lee et al., 2021). Further, Black, compared to Latino and White, youth reported greater perceived risk of harm from alcohol and cannabis use (Johnston et al., 2023), and greater peer disapproval of use (Lee et al., 2021). In early adolescence, perceived friend disapproval plays an important role as a protective factor associated with delayed substance use onset (Field & Prinstein, 2023; Mrug & McCay, 2013). Although studies have documented roles for these risk factors at the population-level, gaps remain regarding their relative importance at the person-level among Black and Latino youth, understudied groups who report similar or higher rates of certain types of substance use (e.g., cannabis) relative to White youth (Miech et al., 2024).

Specifically, in contrast to population-level results, an individual’s report of precursors might not reflect uniformly high or low risk across all precursors (Sartor et al., 2022), or similar levels of risk across substances. To better capture how substance use precursors co-occur at the person-level, in Black and Latino youth, this study used latent class analysis (LCA), a person-centered analytic approach (Spurk et al., 2020). LCA identifies distinct classes or subgroups that share a common pattern of item endorsement at the person-level. Identifying distinct personcentered subgroups can inform tailored prevention efforts based on specific precursor profiles (Bo et al., 2023).

After identifying latent classes, correlates (e.g., sex at birth) of the classes, which might differ by race/ethnicity (Brown et al., 2004), can be used to inform tailored prevention strategies. Correlate selection, based on an ecodevelopmental framework, (Marsiglia & Kiehne, 2020) covers multiple levels: individual, family and peer, and neighborhood conditions (Ayón et al., 2020). At the individual level, sex at birth (Su & Supple, 2014), age (Johnson et al., 2023), socioeconomic status (SES) (Nagata et al., 2023), substance use (Zucker, 2008), importance of religion (Johnson et al., 2023), and externalizing behavior (Miller et al., 2023; Watts et al., 2021) have been associated with substance use risk. At the family level, parental monitoring (Hemovich et al., 2011) and familism, cultural values emphasizing the importance of family commitment, have been associated with lower substance use risk (Sanchez et al., 2023). A key peer factor includes perceived peer norms regarding use, such that perceived peer disapproval of use has been associated with delayed use onset (Mrug & McCay, 2013). At the neighborhood level, poor neighborhood conditions, such as high un-employment and poverty rates (Trucco, 2020) have been associated with substance use risk. Importantly, person-centered analyses can, reveal, for example, the differential importance of familism for Latino youth (Cruz et al., 2019), and the distinct importance of religion for Black youth (Quinn et al., 2023).

In addition to examining latent class correlates, the extent to which latent classes prospectively predict a relevant outcome, such as substance use expectancies, would support their value in guiding prevention (Cruz & Dunn, 2003). Substance use expectancies, or the beliefs about the effects of substance use, robustly predict early initiation in youth (Montes et al., 2019; Smit et al., 2018), and while actual substance use might be low (low baserate outcome), all youth can report expectancies regardless of use (Montes et al., 2019). Greater positive, relative to negative, substance use expectancies consistently predict use onset (Smit et al., 2018), supporting a focus on positive expectancies. In the national Adolescent Brain Cognitive Development (ABCD) Study, Black and Latino, relative to White, youth reported lower positive alcohol expectancies (Nagata et al., 2023; Sanchez et al., 2023), and higher positive expectancies were associated with alcohol sipping (Murphy et al., 2021; Nagata et al., 2023). These emerging findings support further investigation of possible differences among Black and Latino youth in the reltion of substance use precursor classes with positive alcohol and cannabis expectancies over follow-up.

The current analyses used data from the ABCD Study (Garavan et al., 2018). Analyses aimed to identify and characterize co-occurring patterns of key precursors (i.e., perceived ease of access, perceived risk of harm, friends’ disapproval of use) of alcohol and cannabis use in Black and Latino youth, who are less studied, relative to White youth. Identifying latent classes of substance use precursors using a person-centered analytic approach is critical to tailoring prevention, because the impact of a given risk factor on the outcome of interest may depend on cooccurring risk and protective factors (Newcomb & Bentler, 1986). Correlates representing individual, family and peer, and neighborhood-level factors were investigated to gain insight into multi-level influences that can inform tailoring of prevention for specific subgroups. We expected to identify subgroups that were generally low or high on all precursors, as well as additional subgroups that showed, for example, differences for alcohol versus cannabis in access to and perceptions of harm associated with use. We also explored whether classes differed by race/ethnicity.

## 2. Method

### 2.1. Participants and procedure

Analyses used data from the 21-site ABCD Study’s (Garavan et al., 2018) 2-year follow-up (release 5.1; National Institute of Mental Health Data Archive). From 2016 to 2018, the study recruited youth (N = 11,875) aged 9 to 10, primarily through schools (Garavan et al., 2018). At each assessment, youth and their primary caregiver (parent) completed a battery assessing mental and physical health, and cultural and environmental conditions (Barch et al., 2018; Barch et al., 2021; Lisdahl et al., 2018). A centralized Institutional Review Board approved ABCD study protocols. Parents provided written informed consent, and youth provided assent for study participation.

At 2-year follow-up, youth were 10.5–13.5 years old. The ABCD Study categorized race/ethnicity using parent-reported youth race and youth ethnicity (Latino/Hispanic or Non-Hispanic/Latino). All youth identified as Latino/Hispanic ethnicity were categorized as Latino race/ethnicity (N = 2,011). Youth identified as non-Hispanic/Latino ethnicity and “Black” race were categorized as Black race/ethnicity (N = 1,320); a small number (n = 175) identified as both Hispanic/Latino and Black. Youth with missing data on any covariate or outcome (Follow-up 3 positive expectancies) (n = 328) were excluded from analyses (Bakk et al., 2013), resulting in an analysis sample of N = 3,003 (31.0% Black, 69.0% Latino). Attrition analyses indicated no bias due to this exclusion on sex or age.

### 2.2. Measures

Study measures were either completed at baseline or 2-year follow-up, with the exception of the positive expectancies outcomes which were completed at Follow-up 3. Measure descriptions and psychometrics in the ABCD sample have been reported (Barch et al., 2018; Barch et al., 2021; Lisdahl et al., 2018; Zucker et al., 2018). Adjusted scores accounted for bias in measurement across race/ethnicity and sex (Chung et al., 2025; Sartor, Powell, et al., 2025).

#### 2.2.1. Latent class indicators

Latent classes were identified using youth report covering the three domains of (1) perceived ease of access, (2) friends’ disapproval of use, and (3) perceived risk of harm due to use (Lisdahl et al., 2018). Each domain was asked for alcohol (beer, wine or liquor) and cannabis. A total of 6 dichotomous indicators was used (Table 1). *Perceived ease of access* (2 indicators) was queried with the item, “If you wanted to get some [substance] how easy would it be for you to get some?” coded 1= “sort of easy” or “very easy” and 0= “very hard”, “sort of hard” or “don’t know”. *Perceived friends’ disapproval of use* (2 indicators) was assessed with “How do you think your close friends feel (or would feel) about you trying [substance]?” Disapproval was assessed for trying 1–2 drinks, and 1–2 puffs of cannabis, coded 1= “disapprove” or “strongly disapprove” and 0= “not disapprove” or “don’t know”. *Perceived risk of harm due to use* (2 indicators) was queried with “How much do you think people risk harming themselves (physically or in other ways) if they try [substance]? Perceived risk was coded 1= “moderate risk” or “great risk” and 0= “no risk”, “slight risk”, or “don’t know”.

#### 2.2.2. Covariates

##### Individual factors.

Demographics included youth reported age at Follow-up 2, sex at birth, and parent reported race/ethnicity. SES was assessed using parent reported household income (below $50 K: 1 = yes and 0 = no; and above $100 k per year: 1 = yes and 0 = no). Brief Problem Behavior measure (Achenbach & Ruffle, 2000) collected youth reported internalizing and externalizing behavior (ω = 0.83 and 0.73, respectively). Parents reported on importance of religion in their child’s life “In general, how important are your child’s religious and spiritual beliefs in his/ her daily life?” (1=“not at all” to 4=“very”).

##### Substance use factors.

Youth reported on any alcohol or cannabis use (Lisdahl et al., 2018) through 2-year follow-up (none = 0, any use = 1).

##### Family and peer factors.

Youth reported on parental monitoring (Zucker et al., 2018) through four items (e.g., “How often do you talk to your mom/dad or guardian about your plans for the coming day?”, rated 1=”never” to 5=”always”; ω = 0.54). The Mexican American Cultural Values (MACV) measure (Knight et al., 2009) assessed youth reported family support (FS; ω = 0.86), family obligations (FO; ω = 0.79), and family referent (FR; i.e., family as a reference for one’s behavior and decisions; ω = 0.86). The Peer Behavior Profile (Lisdahl et al., 2018) (6 items) includes two subscales on youth involvement with prosocial (e.g., get good grades; ω = 0.45) and delinquent peers (e.g., rule breaking; ω = 0.59), with items rated 1=”none or almost none” to 5=”all or almost all”.

##### Neighborhood factors.

The Area Deprivation Index (ADI) provides a standard indicator of neighborhood disadvantage based on multiple neighborhood-level statistics. ADI values represent national-level percentiles (range: 1–100) with higher percentiles representing greater deprivation. We analyzed ADI quartiles (i.e., ≤25^th^, 26–50, 51–75, ≥76 percentile), see (Sartor, Latendresse, et al., 2025).

#### 2.2.3. Outcomes at 1-year follow-up (ABCD follow-up 3)

##### Positive alcohol and cannabis use expectancies.

The Alcohol Expectancy Questionnaire-Adolescent, Brief (AEQ-AB) (Lisdahl et al., 2018) assessed 4 positive alcohol expectancies (e.g., “alcohol makes a person relax”) rated on a 5-point scale (1 = strongly disagree to 5 = strongly agree; ω = 0.71 at Follow-up 2, ω = 0.74 at Follow-up 3). The Marijuana Effect Expectancy Questionnaire-Brief (MEEQ-B) (Torrealday et al., 2008) assessed 3 positive cannabis use expectancies (e.g., “using cannabis helps a person relax”) rated on a 5-point scale (1 = strongly disagree to 5 = strongly agree; ω = 0.81 at Follow-up 2, ω = 0.83 at Follow-up 3).

### 2.3. Analysis plan

Latent class analysis (LCA) was used to identify distinct subgroups across 3 substance use precursor domains (6 total indicators) using Latent Gold version 6.1 (Vermunt & Magidson, 2016). LCA accounted for nesting of individuals within families and sites (Dick et al., 2021), and accommodated missing data using full information maximum likelihood (Vermunt & Magidson, 2005). LCA tested the fit of 1–9 latent classes, and considered the following in selecting the best fitting model, e.g.: Bayesian Information Criterion [BIC] and Akaike Information Criterion [AIC] (lower value = better fit for both indices (Henson et al., 2007; Spurk et al., 2020)), and avoiding classes with low prevalence (e. g., <5%) (Nylund-Gibson & Choi, 2018). LCA generates estimates of model parameters, such as probabilities of membership in a given class, and item response probabilities that are conditional on class membership.

The adjusted three-step method (Bakk & Vermunt, 2016) examined latent class correlates (simultaneous entry), and prediction of the outcome, positive expectancies one year later, in the same model. In Step 3, separate models were run to predict alcohol and cannabis expectancies outcomes. False discovery rate (FDR) adjustment for multiple testing was used (Leek et al., 2017). Interactions were examined in relation to the latent classes (e.g., race/ethnicity), and prediction of positive expectancies, however, since no interaction was statistically significant, none were included in the final models. For covariates, exploratory post-hoc pairwise comparisons were examined.

## 3. Results

### 3.1. Latent substance use precursor classes

**Fig. 1.**
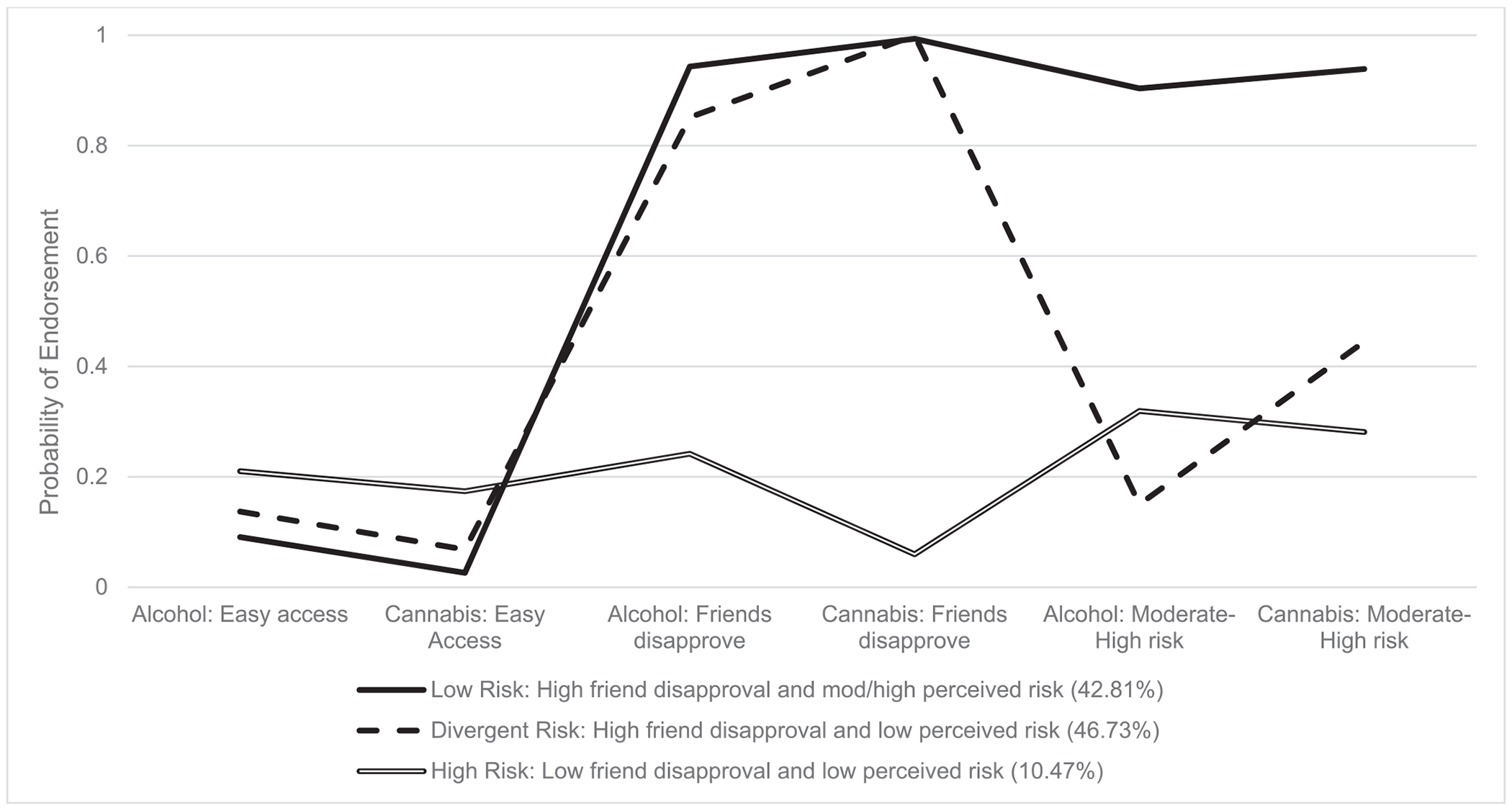
Indicators of access, friend disapproval, and perceived risk of harm by latent precursor class.

Testing the fit of 1–9 classes (Supplemental Table 1) indicated that the 5-class model had the lowest BIC. However, both 5- and 4-class models included classes with only 3% and 4% of cases, respectively, whereas the 3-class model had 10% of cases in the smallest class (Table 1). To avoid classes with low prevalence (e.g., <5% in a class), we selected the 3-class model (Fig. 1). The 3-class solution had good average posterior probabilities for the most likely class assignment (means for class 1 = 0.84, SD = 0.004; class 2 = 0.90, SD = 0.002; class 3 = 0.94, SD = 0.009). Excluding youth who reported lifetime alcohol or cannabis use (which can shape positive expectancies) resulted in similar classes (see Supplemental Material).

All three classes reported low access to alcohol and cannabis. The three classes included High friend disapproval and moderate/high risk of harm (“low risk”, strong protective factors: 42.81%), High friend disapproval but low risk of harm (“divergent”, rather than consistent, precursor endorsement: 46.73%), and Low friend disapproval and low risk of harm (“high risk”, consistent report of risk factors for use: 10.47%). Contrary to prediction, endorsement probabilities for alcohol and cannabis items were similar within each class. The classes differed (*p* < 0.0001) on the six indicators (Table 1), with post-hoc paired comparisons showing consistent differences between low and high risk classes (Supplemental Table 2).

### 3.2. Latent class correlates

Significant differences across latent classes were identified for both the alcohol (Table 2) and cannabis (Table 3) outcome models after FDR adjustment for individual, peer, and family correlates: externalizing, peer rule breaking, parental monitoring and family as referent. The classes did not differ by race/ethnicity. Post-hoc paired comparison results for correlates were similar for the alcohol and cannabis models, indicating differences between low risk vs high risk and divergent classes on externalizing behavior and parental monitoring (Supplemental Tables 3 and 4). Low risk and divergent classes differed from high risk on peer rule breaking. The latent classes also differed on lifetime alcohol use (Table 2), such that the low risk class differed significantly from both the divergent and high risk classes. In contrast, the classes did not differ in lifetime cannabis use (Table 3).

### 3.3. Latent classes as predictors of outcome: positive expectancies one-year later

Overall, the classes differed on both positive alcohol (Table 2) and cannabis (Table 3) expectancies one year later (*p* < 0.0001; all small effects). For positive alcohol expectancies, only low risk and divergent classes differed, with the low risk class reporting lower positive expectancies (Table 2). For cannabis, the low risk class reported lower positive expectancies than the divergent class, and the divergent, relative to the high risk, class reported slightly higher positive expectancies (Table 3; also see Technical note).

## 4. Discussion

These person-centered analyses identified three latent classes of precursors to alcohol and cannabis use in Black and Latino pre-adolescents. In addition to one overall low risk class (High friend disapproval/Mod-High risk of harm), and one overall high risk class (Low friend disapproval/Low risk of harm), a “divergent” class characterized by protective (high friend disapproval) and risk-elevating (low perceived risk of harm) precursors emerged. Class membership was associated with positive alcohol and cannabis expectancies one year later. Classes did not differ by race/ethnicity in this national sample of Black and Latino youth, indicating the importance of shared risk and protective factors in these understudied groups. Shared factors that were differentially associated with the latent classes included individual-level factors (e.g., externalizing behavior), family factors (e.g., parental monitoring, familism), and peer factors (e.g., rule breaking peers). The latent classes add an important new perspective to existing research on the clustering of precursors at the person-level, to inform tailored prevention strategies based on distinct risk profiles in Black and Latino youth.

### 4.1. Latent class indicators

Youth in all three latent classes reported low access to both alcohol and cannabis, which aligns with low levels of lifetime alcohol and cannabis use through Follow-up 2 (0.65% full alcoholic drink, 0.64% tried cannabis) (Sullivan et al., 2022). The limited direct experience with alcohol and cannabis in this early adolescent sample likely explains the similar item endorsement probabilities for alcohol and cannabis variables used to define the classes.

Regarding the domain of perceived peer disapproval, two of the latent classes reported high peer disapproval, but differed in perceived harm due to use (i.e., moderate/high vs low risk). Specifically, the divergent class reported an important “mismatch” between individual (perceived harm) and peer (friend disapproval) precursors of use. That is, an adolescent in the divergent class might, in the future, show a risky shift away from peers who disapprove of use toward peers whose views are more aligned with the current perception of low risk of harm from use. Alternatively, an adolescent in the divergent class might show a protective shift away from their risky perception of low risk of harm to spending more time with their peers who currently disapprove of use. Regarding prevention implications, a potential modifiable target for the divergent class includes addressing the “misperception” of low risk of harm and strengthening relations with peers who disapprove of use (Catalano et al., 2012).

In determining correlates of the classes, the classes were associated with individual, family and peer factors. Consistent with prior research, the low and high risk classes were distinguished by individual-level factors of lifetime alcohol use (Zucker, 2008) and externalizing behavior (Miller et al., 2023; Watts et al., 2021); peer factors such as deviant peers (Watts et al., 2024); and family factors such as parental monitoring (Hemovich et al., 2011) and family as referent (Sanchez et al., 2023). Notably, the low risk and divergent classes differed on endorsement of family as referent, with the low risk class endorsing this facet of familism to a greater extent, which could protect youth against substance use (Cruz et al., 2019; Gonzales et al., 2017). Critically, this study adds to population-based research by providing a novel person-centered perspective on precursors and risk. Identifying distinct subgroups with specific individual-level risk and protection profiles enables delivery of personalized prevention strategies (Collins et al., 2004).

Prevention strategies tailored to risk profile (Collins et al., 2004) could, for example, for the low risk class, maintain this class’s strengths in their commitment to family (i.e., family as referent), and low levels of externalizing behavior. By comparison, personalized prevention for the divergent class might address their misconceptions regarding perceived risk of harm due to use and strengthen supportive family bonds to reduce affiliation with deviant peers (Catalano et al., 2012). The high risk class might benefit from early, developmentally tailored intervention (Catalano et al., 2012), since some youth in this class already experimented with alcohol.

### 4.2. Latent classes predicted positive alcohol and cannabis expectancies one year later

The finding that the classes predicted positive expectancies one year later adds new prospective information to prior cross-sectional ABCD results (Nagata et al., 2023; Sanchez et al., 2023). The person-centered results also complement prior population-based results (Montes et al., 2019; Smit et al., 2018). In this study, for both alcohol and cannabis, the low risk (vs divergent) class reported lower positive expectancies; and for cannabis, the divergent (vs high risk) class reported slightly higher positive expectancies (all small effects). Notably, analyses did not indicate that low and high risk classes differed, likely due to factors such as the proportionally small high risk class size and the high risk class’s relatively large SEs, along with other factors (e.g., uncertainty in class assignment) (Gudicha et al., 2017). Importantly, the ability to detect a significant difference between the high risk and divergent classes (e.g., for positive cannabis expectancies) was likely determined by the SE of the difference for the pairwise contrast (not by class-specific SEs alone; see Technical Note) (Gudicha et al., 2017). Thus, for positive cannabis (vs alcohol) expectancies, there was greater precision to detect a group difference (e.g., high risk vs divergent classes). The predictive validity of the identified latent classes suggests their value in identifying substance use risk for personalized prevention delivery.

## 5. Limitations

Analyses focused on Black and Latino youth since these populations are understudied and at-risk for substance use (Keyes et al., 2022). There is heterogeneity within these broad racial/ethnic groupings that was not captured, and merits attention. Results are limited in generalizability to Black and Latino early adolescents with limited lifetime alcohol and cannabis use. Analyses focused on positive (vs negative) expectancies. Although the 3-class model showed the best fit, a measure of model fit, entropy, was relatively low, indicating caution in interpreting results, and the importance of replication. Some omegas are below a standard cut-off, possibly due to scale brevity. Other neighborhood variables (e. g., community resources) and perceived peer characteristics (e.g., approval of use, actual use) warrant study.

## 6. Conclusions

These person-centered analyses identified 3 latent classes of substance use precursors that predicted positive alcohol and cannabis expectancies one year later in the understudied groups of Black and Latino youth. The low risk (High friend disapproval/moderate-high risk of harm), divergent (High friend disapproval/ low risk of harm), and high risk (Low friend disapproval/ low risk of harm) classes were distinguished by individual, and family and peer factors that can inform personalized tailoring of multi-level prevention strategies.

## Supplementary Material

Sartor Chung supp

## Figures and Tables

**Table 1:** Three latent classes of alcohol and cannabis precursors: Indicators by class.

	Total Sample N = 3,003	“Low risk” High friend disapproval and high perceived risk (42.81%)	“Divergent risk” High friend disapproval and low perceived risk (46.73%)	“High risk” Low friend disapproval and low perceived risk (10.47%)	Wald statistic	*p*-value	Significant paired comparisons
**Easy to access (%)**							
Alcohol	12.48	9.08	13.70	21.02	22.77	*p* < 0.0001	L ≠ H, D ≠ H
Cannabis	6.11	2.60	6.82	17.38	48.69	*p* < 0.0001	L ≠ D, L ≠ H, D ≠ H
**Friends disapprove of use (%)**							
Alcohol	82.62	94.33	85.13	24.16	355.49	*p* < 0.0001	L ≠ D, L ≠ H, D ≠ H
Cannabis	89.65	99.35	99.94	5.97	50.43	*p* < 0.0001	L ≠ H, D ≠ H
**Moderate to high risk of harm due to use (%)**							
Alcohol	49.56	90.35	15.01	31.93	21.29	*p* < 0.0001	L ≠ D, D ≠ H
Cannabis	64.20	93.88	44.49	28.10	31.48	*p* < 0.0001	L ≠ D, L ≠ H, D ≠ H

Notes: L (Low risk) = High friend disapproval and low perceived risk of harm; D (Divergent risk) = High friend disapproval and high perceived risk of harm; H (High risk) = Low friend disapproval and low perceived risk of harm.

**Table 2:** Alcohol expectancies model: Latent class correlates (conditional estimates).

	Total Sample N = 3,003	“Low risk” High friend disapproval and high perceived risk (42.81%)	“Divergent risk” High friend disapproval and low perceived risk (46.73%)	“High risk” Low friend disapproval and low perceived risk (10.47%)	Wald statistic	*p*-value	Significant paired comparisons
**Individual Factors**							
Age (mean, SE)	12.00 (0.01)	11.95 (0.02)	12.04 (0.02)	12.04 (0.05)	2.19 (2)	0.33	
Sex: Female (%)	49.61	48.00	52.16	44.76	4.60 (2)	0.10	
Black (%)	31.00	31.79	29.98	32.34	0.02 (2)	0.99	
Household income below $50 K/year (%)	63.48	64.10	61.32	70.46	1.43 (2)	0.49	
Household income ≥$100 K/year (%)	10.69	9.13	13.08	6.50	3.94 (2)	0.14	
Internalizing (mean, SE)	0.08 (0.02)	0.03 (0.03)	0.09 (0.03)	0.21 (0.06)	4.32 (2)	0.11	
Externalizing (mean, SE)	0.08 (0.02)	−0.08 (0.03)	0.15 (0.03)	0.36 (0.06)	20.53 (2)	*p* < 0.0001	L ≠ D, D ≠ H
Importance of religion (mean, SE)	3.02 (0.02)	3.10 (0.04)	2.98 (0.03)	2.85 (0.08)	3.56 (2)	0.17	
**Substance Use**							
Any alcohol use through Follow-up 2 (%)	21.33	14.55	25.17	31.88	21.92 (2)	*p* < 0.0001	L ≠ D, D ≠ H
	Total Sample N=3,003	“Low risk” High friend disapproval and high perceived risk (42.81%)	“Divergent risk” High friend disapproval and low perceived risk (46.73%)	“High risk” Low friend disapproval and low perceived risk (10.47%)	Wald statistic	*p*-value	Significant paired comparisons

**Family and Peer factors**							
Parental monitoring (mean, SE)	−0.06 (0.02)	0.08 (0.03)	−0.12 (0.03)	−0.42 (0.07)	15.39 (2)	*p*<.0001	L≠D, D≠H
MACV FS (mean, SE)	0.12 (0.02)	0.31 (0.03)	0.01 (0.03)	−0.21 (0.06)	1.85 (2)	0.40	
MACV FO (mean, SE)	0.10 (0.02)	0.27 (0.03)	0.01 (0.03)	−0.23 (0.06)	5.51 (2)	0.06	
MACV FR (mean, SE)	0.24 (0.02)	0.47 (0.03)	0.10 (0.03)	−0.01 (0.06)	20.73 (2)	*p*<.0001	L≠D
Peer prosocial behavior (mean, SE)	−0.11 (0.01)	−0.08 (0.02)	−0.14 (0.02)	−0.10 (0.05)	0.63 (2)	0.73	
Peer rule breaking (mean, SE)	0.16 (0.01)	0.06 (0.03)	0.16 (0.02)	0.52 (0.06)	24.69 (2)	*p*<.0001	L≠H, D≠H
**Neighborhood factors**							
ADI 2nd quartile	29.05	29.65	29.80	23.31	0.89 (2)	0.64	
ADI 3rd quartile	21.33	20.68	21.19	24.61	1.23 (2)	0.54	
ADI 4th quartile	25.67	25.18	25.02	30.61	1.11 (2)	0.57	
	Total Sample N=3,003	“Low risk” High friend disapproval and high perceived risk (42.81%)	“Divergent risk” High friend disapproval and low perceived risk (46.73%)	“High risk” Low friend disapproval and low perceived risk (10.47%)	Wald statistic	*p*-value	Significant paired comparisons

**Outcome: Follow-up 3**							
Alcohol Positive Expectancies (mean, SE)	−0.05 (0.02)	−0.22 (0.03)	0.08(0.03)	0.07(0.06)	15.29 (2)	*p*<.0001	L≠D

Notes: SE= Standard error; $50 K= $50,000; $100 K=$100,000; MACV = Mexican American Cultural Values, FR = Family referent, FO = Family obligations, FS = Family support; L (Low risk) = High friend disapproval and low perceived risk of harm; D (Divergent risk) = High friend disapproval and high perceived risk of harm; H (High risk) = Low friend disapproval and low perceived risk of harm.

False discovery rate (FDR) adjustment for multiple testing was used; original *p*-values ≤ 0.016 are considered significant at FDR *p* < 0.05.

Regarding post-hoc paired comparison for Alcohol Positive Expectancies: While the mean parameter estimates for the alcohol positive expectancies outcome appear to be similar for the “Divergent risk” (0.08) and “High risk” (0.07) classes, the Wald statistic for the post-hoc pairwise comparison between the “Low risk” vs “Divergent risk” class is greater (Wald = 15.04, df = 1, *p* = 0.0001), than the Wald statistic for the post-hoc pairwise comparison between the “Low risk” vs “High risk” class (Wald = 4.43, df = 1, *p* = 0.035, which fell short of the *p* < 0.016 cut-off for FDR adjusted statistical significance at *p* < 0.05), likely due, in part, to being underpowered (smaller sample size in the “High risk” class).

**Table 3:** Cannabis expectancies model: Latent class correlates (conditional estimates).

	Total Sample N = 3,003	“Low risk” High friend disapproval and high perceived risk (42.81%)	“Divergent risk” High friend disapproval and low perceived risk (46.73%)	“High risk” Low friend disapproval and low perceived risk (10.47%)	Wald statistic	*p*-value	Significant paired comparisons
**Individual Factors**							
Age (mean, SE)	12.00 (0.01)	11.95 (0.02)	12.04 (0.02)	12.04 (0.05)	3.07 (2)	0.22	
Sex: Female (%)	49.61	48.00	52.16	44.76	3.81 (2)	0.15	
Black (%)	31.00	31.79	29.98	32.34	0.28 (2)	0.87	
Household income below $50 K/year (%)	63.48	64.10	61.32	70.46	0.93 (2)	0.63	
Household income ≥$100 K/year (%)	10.69	9.13	13.08	6.50	4.25 (2)	0.12	
Internalizing (mean, SE)	0.08 (0.02)	0.03 (0.03)	0.09 (0.03)	0.21 (0.06)	3.74 (2)	0.15	
Externalizing (mean, SE)	0.08 (0.02)	−0.08 (0.03)	0.15 (0.03)	0.36 (0.06)	23.25 (2)	*p* < 0.0001	L ≠ D, D ≠ H
Importance of religion (mean, SE)	3.02 (0.02)	3.10 (0.04)	2.98 (0.03)	2.85 (0.08)	4.23 (2)	0.12	
**Substance Use**							
Any alcohol use through Follow-up 2 (%)	0.34	0.09	0.10	2.45	6.35 (2)	0.04	
	Total Sample N=3,003	“Low risk” High friend disapproval and high perceived risk (42.81%)	“Divergent risk” High friend disapproval and low perceived risk (46.73%)	“High risk” Low friend disapproval and low perceived risk (10.47%)	Wald statistic	*p*-value	Significant paired comparisons

**Family and Peer factors**							
Parental monitoring (mean, SE)	−0.06 (0.02)	0.08 (0.03)	−0.12 (0.03)	−0.42 (0.07)	16.69 (2)	*p*<.0001	L≠D, D≠H
MACV FS (mean, SE)	0.12 (0.02)	0.31 (0.03)	0.01 (0.03)	−0.21 (0.06)	1.60 (2)	0.45	
MACV FO (mean, SE)	0.10 (0.02)	0.27 (0.03)	0.01 (0.03)	−0.23 (0.06)	6.12 (2)	0.05	
MACV FR (mean, SE)	0.24 (0.02)	0.47 (0.03)	0.10 (0.03)	−0.01 (0.06)	21.95 (2)	*p*<.0001	L≠D
Peer prosocial behavior (mean, SE)	−0.11 (0.01)	−0.08 (0.02)	−0.14 (0.02)	−0.10 (0.05)	0.77 (2)	0.68	
Peer rule breaking (mean, SE)	0.16 (0.01)	0.06 (0.03)	0.16 (0.02)	0.52 (0.06)	22.38 (2)	*p*<.0001	L≠H, D≠H
**Neighborhood factors**							
ADI 2nd quartile	29.05	29.65	29.80	23.31	1.08 (2)	0.58	
ADI 3rd quartile	21.33	20.68	21.19	24.61	0.93 (2)	0.63	
ADI 4th quartile	25.67	25.18	25.02	30.61	0.92 (2)	0.63	
	Total Sample N=3,003	“Low risk” High friend disapproval and high perceived risk (42.81%)	“Divergent risk” High friend disapproval and low perceived risk (46.73%)	“High risk” Low friend disapproval and low perceived risk (10.47%)	Wald statistic	*p*-value	Significant paired comparisons
**Outcome: Follow-up 3**							
Cannabis Positive Expectancies (mean, SE)	−0.05 (0.02)	−0.26 (0.03)	0.11 (0.03)	0.10 (0.07)	29.08 (2)	*p*<.0001	L≠D, D≠H

Notes: SE= Standard error; $50 K= $50,000; $100 K=$100,000; MACV = Mexican American Cultural Values, FR = Family referent, FO = Family obligations, FS = Family support; L (Low risk) = High friend disapproval and low perceived risk of harm; D (Divergent risk) = High friend disapproval and high perceived risk of harm; H (High risk) = Low friend disapproval and low perceived risk of harm. False discovery rate (FDR) adjustment for multiple testing was used; original *p*-values ≤ 0.016 are considered significant at FDR *p* < 0.05.

## Data Availability

The authors do not have permission to share data.

## Appendix A. Supplementary data

Supplementary data to this article can be found online at https://doi.org/10.1016/j.addbeh.2026.108702.

## References

[R1] Achenbach TM, & Ruffle TM (2000). The child behavior checklist and related forms for assessing behavioral/emotional problems and competencies. Pediatrics in Review, 21(8), 265–271. 10.1542/pir.21-8-265 10922023

[R2] Ahuja M, Haeny AM, Sartor CE, & Bucholz KK (2022). Perceived racial and social class discrimination and cannabis involvement among Black youth and young adults. Drug and Alcohol Dependence, 232, Article 109304. 10.1016/j.drugalcdep.2022.109304 PMC10228548 35124388

[R3] Ayón C, Nieri T, & Ruano E (2020). Ethnic-racial socialization among latinx families: A systematic review of the literature. Social Service Review, 94(4), 693–747. 10.1086/712413

[R4] Bakk Z, Tekle FB, & Vermunt JK (2013). Estimating the association between latent class membership and external variables using bias-adjusted three-step approaches. Sociological Methodology, 43(1), 272–311. 10.1177/0081175012470644

[R5] Bakk Z, & Vermunt JK (2016). Robustness of stepwise latent class modeling with continuous distal outcomes. Structural Equation Modeling, 23, 20–31. 10.1080/10705511.2014.955104

[R6] Barch DM, Albaugh MD, Avenevoli S, Chang L, Clark DB, Glantz MD, Hudziak JJ, Jernigan TL, Tapert SF, Yurgelun-Todd D, Alia-Klein N, Potter AS, Paulus MP, Prouty D, Zucker RA, & Sher KJ (2018). Demographic, physical and mental health assessments in the adolescent brain and cognitive development study: Rationale and description. Developmental Cognitive Neuroscience, 32, 55–66. 10.1016/j.dcn.2017.10.010 29113758 PMC5934320

[R7] Barch DM, Albaugh MD, Baskin-Sommers A, Bryant BE, Clark DB, Dick AS, Feczko E, Foxe JJ, Gee DG, Giedd J, Glantz MD, Hudziak JJ, Karcher NR, LeBlanc K, Maddox M, McGlade EC, Mulford C, Nagel BJ, Neigh G, …, Xie L (2021). Demographic and mental health assessments in the adolescent brain and cognitive development study: Updates and age-related trajectories. Developmental Cognitive Neuroscience, 52, Article 101031. 10.1016/j.dcn.2021.101031 PMC8579129 34742018

[R8] Bo A, Goings TC, Evans CBR, Sharma A, Jennings Z, Durand B, Bardeen A, & Murray-Lichtman A (2023). Culturally sensitive prevention programs for substance use among adolescents of color: A systematic review and meta-analysis of randomized controlled trials. Clinical Psychology Review, 99, Article 102233. 10.1016/j.cpr.2022.102233 PMC9847495 36495737

[R9] Boyas JF, Villarreal-Otálora T, & Marsiglia FF. (2019). Alcohol use among latinx early adolescents: Exploring the role of the family. Journal of Alcohol and Drug Education, 63(2), 35–58. 31680706 PMC6824262

[R10] Brown TL, Miller JD, & Clayton RR (2004). The generalizability of substance use predictors across racial groups. The Journal of Early Adolescence, 24(3), 274–302. 10.1177/0272431604265677

[R11] Catalano RF, Fagan AA, Gavin LE, Greenberg MT, Irwin CE Jr., Ross DA, & Shek DT (2012). Worldwide application of prevention science in adolescent health. Lancet, 379(9826), 1653–1664. 10.1016/s0140-6736(12)60238-4 22538180 PMC4398056

[R12] Chung T, Latendresse SJ, Kennelly N, Powell MZ, & Sartor CE (2025). Adjusting for measurement bias in the MEEQ-B across sex and race/ethnicity in the ABCD study. Journal of Studies on Alcohol and Drugs, 86(5), 683–693. 10.15288/jsad.24-00201. 39630417 PMC12419513

[R13] Collins LM, Murphy SA, & Bierman KL (2004). A conceptual framework for adaptive preventive interventions. Prevention Science, 5(3), 185–196. 10.1023/b:prev.0000037641.26017.00 15470938 PMC3544191

[R14] Cruz IY, & Dunn ME (2003). Lowering risk for early alcohol use by challenging alcohol expectancies in elementary school children. Journal of Consulting and Clinical Psychology, 71(3), 493–503. 10.1037/0022-006x.71.3.493 12795573

[R15] Cruz RA, Mechammil M, & Robins RW (2019). Familism, sibling relationship qualities, and sibling sex constellation as predictors of alcohol use among Mexican-origin adolescents. Journal of Family Psychology, 33(7), 868–875. 10.1037/fam0000531 30907607

[R16] Dick AS, Lopez DA, Watts AL, Heeringa S, Reuter C, Bartsch H, Fan CC, Kennedy DN, Palmer C, Marshall A, Haist F, Hawes S, Nichols TE, Barch DM, Jernigan TL, Garavan H, Grant S, Pariyadath V, Hoffman E, …, Thompson WK (2021). Meaningful associations in the adolescent brain cognitive development study. NeuroImage, 239, Article 118262. 10.1016/j.neuroimage.2021.118262 PMC8803401 34147629

[R17] Field NH, Prinstein MJ. Reconciling multiple sources of influence: Longitudinal associations among perceived parent, closest friend, and popular peer injunctive norms and adolescent substance use. Child Dev. 2023 Jul-Aug;94(4):809–825. doi: 10.1111/cdev.13898. Epub 2023 Feb 13. 36779425 PMC10293111

[R18] Garavan H, Bartsch H, Conway K, Decastro A, Goldstein RZ, Heeringa S, Jernigan T, Potter A, Thompson W, & Zahs D (2018). Recruiting the ABCD sample: Design considerations and procedures. Developmental Cognitive Neuroscience, 32, 16–22. 10.1016/j.dcn.2018.04.004 29703560 PMC6314286

[R19] Gonzales NA, Liu Y, Jensen M, Tein JY, White RMB, & Deardorff J (2017). Externalizing and internalizing pathways to Mexican American adolescents’ risk taking. Development and Psychopathology, 29(4), 1371–1390. 10.1017/s0954579417000323 28367763 PMC5575951

[R20] Gudicha DW, Schmittmann VD, & Vermunt JK (2017). Statistical power of likelihood ratio and Wald tests in latent class models with covariates. Behavior Research Methods, 49(5), 1824–1837. 10.3758/s13428-016-0825-y 28039681 PMC5628195

[R21] Hemovich V, Lac A, & Crano WD (2011). Understanding early-onset drug and alcohol outcomes among youth: The role of family structure, social factors, and interpersonal perceptions of use. Psychology, Health & Medicine, 16(3), 249–267. 10.1080/13548506.2010.532560 PMC3088114 21491334

[R22] Henson JM, Reise SP, & Kim KH (2007). Detecting mixtures from structural model differences using latent variable mixture modeling: A comparison of relative model fit statistics. Structural Equation Modeling, 14, 202–226. 10.1080/10705510709336744

[R23] Johnson EC, Paul SE, Baranger DAA, Hatoum AS, Colbert SMC, Lin S, Wolff R, Gorelik AJ, Hansen I, Karcher NR, Bogdan R, & Agrawal A (2023). Characterizing Alcohol Expectancies in the ABCD Study: Associations with Sociodemographic Factors, the Immediate Social Environment, and Genetic Propensities. Behavior Genetics, 53(3), 265–278. 10.1007/s10519-023-10133-2 36662388 PMC10159951

[R24] Johnston LD, Miech RA, Patrick ME, O’Malley PM, Schulenberg JE, & Bachman JG (2023). Monitoring the Future national survey results on drug use 1975-2022: Overview, key findings on adolescent drug use. University of Michigan: Institute for Social Research.

[R25] Keyes KM, Kaur N, Kreski NT, Chen Q, Martins SS, Hasin D, Olfson M, & Mauro PM (2022). Temporal trends in alcohol, cannabis, and simultaneous use among 12th-grade U.S. adolescents from 2000 to 2020: Differences by sex, parental education, and race and ethnicity. Alcohol Clin Exp Res, 46(9), 1677–1686. 10.1111/acer.14914 36125706 PMC9635013

[R26] Knight GP, Gonzales NA, Saenz DS, Bonds DD, Germán M, Deardorff J, Roosav MW, & Updegraff KA (2009). The mexican american cultural values scale for adolescents and adults. The Journal of Early Adolescence, 30(3), 444–481. 10.1177/0272431609338178 PMC2904976 20644653

[R27] Lee MH, Kim-Godwin YS, & Hur H (2021). Race/ethnicity differences in risk and protective factors for marijuana use among U.S. adolescents. BMC Public Health, 21 (1), 1167. 10.1186/s12889-021-11159-z 34193108 PMC8247234

[R28] Leek J, McShane BB, Gelman A, Colquhoun D, Nuijten MB, & Goodman SN (2017). Five ways to fix statistics. Nature, 551(7682), 557–559. 10.1038/d41586-017-07522-z 29189798

[R29] Lisdahl KM, Sher KJ, Conway KP, Gonzalez R, Feldstein Ewing SW, Nixon SJ, Tapert S, Bartsch H, Goldstein RZ, & Heitzeg M (2018). Adolescent brain cognitive development (ABCD) study: Overview of substance use assessment methods. Developmental Cognitive Neuroscience, 32, 80–96. 10.1016/j.dcn.2018.02.007 29559216 PMC6375310

[R30] Marsiglia FF, & Kiehne E (2020). Substance use among Latinx adolescents in the USA: Scope, theory, interventions, and next steps. In Martínez AD, & Rhodes SD (Eds.), New and Emerging Issues in Latinx Health (pp. 97–126). Springer International Publishing.

[R31] Merianos AL, Rosen BL, Montgomery L, Barry AE, & Smith ML (2017). Impact of Perceived Risk and Friend Influence on Alcohol and Marijuana Use among students. The Journal of School Nursing, 33(6), 446–455. 10.1177/1059840517717591 28675076

[R32] Miech RA, Johnston LD, Patrick ME, & O’Malley PM (2024). Monitoring the Future national survey results on drug use 1975–2023: Overview and detailed results for secondary school students. Institute for Social Research.

[R33] Miller AP, Baranger DAA, Paul SE, Hatoum AS, Rogers C, Bogdan R, & Agrawal A (2023). Characteristics associated with cannabis use initiation by late childhood and early adolescence in the adolescent brain cognitive development (ABCD) study. JAMA Pediatrics, 177(8), 861–863. 10.1001/jamapediatrics.2023.1801 37358866 PMC10294012

[R34] Montes KS, Witkiewitz K, Pearson MR, & Leventhal AM (2019). Alcohol, tobacco, and marijuana expectancies as predictors of substance use initiation in adolescence: A longitudinal examination. Psychology of Addictive Behaviors, 33(1), 26–34. 10.1037/adb0000422 30407027 PMC6367043

[R35] Mrug S, & McCay R (2013). Parental and peer disapproval of alcohol use and its relationship to adolescent drinking: Age, gender, and racial differences. Psychology of Addictive Behaviors, 27(3), 604–614. 10.1037/a0031064 23276323 PMC4004115

[R36] Murphy MA, Dufour SC, & Gray JC (2021). The association between child alcohol sipping and alcohol expectancies in the ABCD study. Drug and Alcohol Dependence, 221, Article 108624. 10.1016/j.drugalcdep.2021.108624 33676072

[R37] Nagata JM, Zamora G, Smith N, Sajjad OM, Shim J, Ganson KT, Testa A, & Jackson DB (2023). Social epidemiology of early adolescent alcohol expectancies. BMC Public Health, 23(1), 2502. 10.1186/s12889-023-17434-5 38093235 PMC10720177

[R38] Nawi AM, Ismail R, Ibrahim F, Hassan MR, Manaf MRA, Amit N, Ibrahim N, & Shafurdin NS (2021). Risk and protective factors of drug abuse among adolescents: A systematic review. BMC Public Health, 21(1), 2088. 10.1186/s12889-021-11906-2 34774013 PMC8590764

[R39] Newcomb MD, & Bentler PM (1986). Substance use and ethnicity: Differential impact of peer and adult models. The Journal of Psychology, 120(1), 83–95. 10.1080/00223980.1986.9712618 3488395

[R40] Nylund-Gibson K, & Choi AY (2018). Ten frequently asked questions about latent class analysis. Translational Issues in Psychological Science, 4(4), 440–461. 10.1037/tps0000176

[R41] Quinn CR, Waller B, Hughley A, Boyd D, Cobb R, Hardy K, Radney A, & Voisin DR (2023). The relationship between religion, substance misuse, and mental health among black youth. Religions, 14(3), 325. https://www.mdpi.com/2077-1444/14/3/325. 38009108 10.3390/rel14030325 PMC10673626

[R42] Sanchez M, Gonzalez MR, Fernandez A, Barton A, Diaz V, & Wang W (2023). Sociocultural influences on alcohol expectancies in early adolescence: Findings from the ABCD study. Health Psychology, 42(12), 842–855. 10.1037/hea0001290 37227824 PMC10674043

[R43] Sartor CE, Latendresse SJ, Jackson KM, Steers MN, Lipperman-Kreda S, Slade T, & Chung T (2025). Parents’ perspectives and behaviors regarding their child’s access to alcohol: Variation by race/ethnicity, socioeconomic status, and neighborhood. Alcohol Clin Exp Res (Hoboken), 49(1), 234–243. 10.1111/acer.15498 39701594 PMC11740169

[R44] Sartor CE, Powell MZ, Kennelly N, Chung T, & Latendresse SJ (2025). Establishing measurement equivalence across sex, race/ethnicity, and intersectional identity for the alcohol expectancy questionnaire-adolescent, brief: findings from the ABCD study. Alcohol and Alcoholism, 60(4). 10.1093/alcalc/agaf039 PMC12685248 40561462

[R45] Sartor CE, Ye F, Simon P, Zhai ZW, Hipwell AE, & Chung T (2022). Cross-substance patterns of alcohol, cigarette, and cannabis use initiation in Black and White adolescent girls. Preventive Medicine, 156, Article 106979. 10.1016/j.ypmed.2022.106979 PMC8922285 35124100

[R46] Smit K, Voogt C, Hiemstra M, Kleinjan M, Otten R, & Kuntsche E (2018). Development of alcohol expectancies and early alcohol use in children and adolescents: A systematic review. Clinical Psychology Review, 60, 136–146. 10.1016/j.cpr.2018.02.002 29449029

[R47] Spurk D, Hirschi A, Wang M, Valero D, & Kauffeld S (2020). Latent profile analysis: A review and “how to” guide of its application within vocational behavior research. J Vocational Behavior, 120, Article 103445. 10.1016/j.jvb.2020.103445

[R48] Su J, & Supple AJ (2014). Parental, peer, school, and neighborhood influences on adolescent substance use: direct and indirect effects and ethnic variations. Journal of Ethnicity in Substance Abuse, 13(3), 227–246. 10.1080/15332640.2013.847393 25176117

[R49] Sullivan RM, Wade NE, Wallace AL, Tapert SF, Pelham WE 3rd, Brown SA, Cloak CC, Feldstein Ewing SW, Madden PAF, Martz ME, Ross JM, Kaiver CM, Wirtz HG, Heitzeg MM, & Lisdahl KM (2022). Substance use patterns in 9 to 13-year-olds: longitudinal findings from the adolescent brain cognitive development (ABCD) study. Drug and Alcohol Dependence Reports, 5. 10.1016/j.dadr.2022.100120 PMC9850746 36687306

[R50] Torrealday O, Stein LA, Barnett N, Golembeske C, Lebeau R, Colby SM, & Monti PM (2008). Validation of the marijuana effect expectancy questionnaire-brief. Journal of Child & Adolescent Substance Abuse, 17(4), 1–17. 10.1080/15470650802231861 22058648 PMC3207500

[R51] Trucco EM (2020). A review of psychosocial factors linked to adolescent substance use. Pharmacology Biochemistry and Behavior, 196, Article 172969. 10.1016/j.pbb.2020.172969 PMC7415605 32565241

[R52] Vermunt J, & Magidson J (2016). Upgrade Manual for Latent GOLD 5.1 Statistical Innovations Inc.

[R53] Vermunt JK, & Magidson J (2005). Latent Variable. In Everitt B, & Howell D (Eds.), Encyclopedia of Statistics in Behavioral Science. Inc: John Wiley & Sons. 10.1002/0470013192.bsa339.

[R54] Watts AL, Wood PK, Jackson KM, Lisdahl KM, Heitzeg MM, Gonzalez R, Tapert SF, Barch DM, & Sher KJ (2021). Incipient Alcohol Use in Childhood: Early Alcohol Sipping and its Relations with Psychopathology and Personality - Corrigendum. Development and Psychopathology, 33(3), 1139. 10.1017/s0954579420001170 33043878

[R55] Watts LL, Hamza EA, Bedewy DA, & Moustafa AA (2024). A meta-analysis study on peer influence and adolescent substance use. Current Psychology, 43(5), 3866–3881. 10.1007/s12144-023-04944-z

[R56] Zucker RA (2008). Anticipating problem alcohol use developmentally from childhood into middle adulthood: What have we learned? Addiction, 103 Suppl 1(Suppl 1), 100–108. 10.1111/j.1360-0443.2008.02179.x 18426543 PMC2593849

[R57] Zucker RA, Gonzalez R, Feldstein Ewing SW, Paulus MP, Arroyo J, Fuligni A, Morris AS, Sanchez M, & Wills T (2018). Assessment of culture and environment in the Adolescent Brain and Cognitive Development Study: Rationale, description of measures, and early data. Developmental Cognitive Neuroscience, 32, 107–120. 10.1016/j.dcn.2018.03.004 29627333 PMC6436615

