# Supplementary material for: Latent classes of substance use precursors predict positive alcohol and cannabis expectancies one year later in Black and Latino early adolescents in the ABCD study
Source: Addict Behav. Author manuscript; Available in PMC 2026 Aug 1. (PMC13255241; doi:10.1016/j.addbeh.2026.108702)
Supplement: Sartor Chung supp [file NIHMS2177795-supplement-Sartor_Chung_supp.docx]

Supplemental Table 1. Comparison of model fit: testing 1-9 class solutions

| **Classes** | **LL** | **BIC(LL)** | **AIC(LL)** | **N par** | **L²** | **df** | ***p*-value** | **Class. Err.** | **Entropy R²** |
| --- | --- | --- | --- | --- | --- | --- | --- | --- | --- |
| 1 | -8537.47 | 17123.60 | 17086.93 | 6 | 1651.97 | 90 | *p*<.00001 | 0 | 1 |
| 2 | -8064.48 | 16234.40 | 16154.95 | 13 | 705.99 | 83 | *p*<.00001 | 0.1361 | 0.5266 |
| 3 | -7868.38 | 15898.98 | 15776.76 | 20 | 313.80 | 76 | *p*<.00001 | 0.1253 | 0.6501 |
| 4 | -7803.91 | 15826.81 | 15661.81 | 27 | 184.85 | 69 | *p*<.00001 | 0.1221 | 0.6788 |
| 5 | -7762.71 | **15801.20** | 15593.43 | 34 | 102.47 | 62 | 0.00094 | 0.1198 | 0.6913 |
| 6 | -7748.89 | 15830.33 | 15579.78 | 41 | 74.82 | 55 | 0.039 | 0.1169 | 0.7105 |
| 7 | -7739.47 | 15868.27 | 15574.94 | 48 | 55.98 | 48 | 0.20 | 0.1489 | 0.6510 |
| 8 | -7735.29 | 15916.69 | 15580.58 | 55 | 47.62 | 41 | 0.22 | 0.2660 | 0.5470 |
| 9 | -7732.56 | 15968.00 | 15589.12 | 62 | 42.16 | 34 | 0.16 | 0.2504 | 0.5462 |

| **Classes** | **LRT (df=7)** | **LRT**  ***p*-value** | **VLRT** | **VLRT**  ***p*-value** | **Bootstrap *p-*value** |
| --- | --- | --- | --- | --- | --- |
| 1 | 945.98 | <.01 |  |  |  |
| 2 | 392.19 | <.01 | 945.98 | <.01 | 0 |
| 3 | 128.95 | <.01 | 392.19 | <.01 | 0 |
| 4 | 82.38 | <.01 | 128.95 | <.01 | 0 |
| 5 | 27.65 | <.01 | 82.38 | <.01 | 0 |
| 6 | 18.84 | <.01 | 27.65 | <.01 | 0.022 |
| 7 | 8.36 | >.05 (ns) | 18.84 | 0.02 | 0.39 |
| 8 | 5.47 | >.05 (ns) | 8.36 | 0.08 (ns) | 0.226 |
| 9 | 945.98 | <.01 | 5.47 | 0.28 (ns) | 0.676 |

Notes: Bolded value=lowest BIC. LL=Log Likelihood, BIC=Bayesian Information Criterion, AIC=Akaike Information

Criterion, N Par= Number of Parameters, L^2^= Log Likelihood^2^, df=degrees of freedom, Class Err= Classification error, LRT=Likelihood Ratio Test, VLRT=Vuong-Lo-Rubin Test, Bootstap Likelihood Ratio Test (BLRT)

Supplemental Table 2. Paired comparisons between the 3 latent classes on the 6 indicators used to define the classes

| **Indicator** | **Paired class comparison** | **Wald (df)** | ***p*-value** |
| --- | --- | --- | --- |
| Ease of access to alcohol | L vs. D * | 5.77 (1) | .016 |
|  | L vs. H * | 6.03 (1) | .014 |
|  | D vs. H * | 22.67 (1) | *p*<0.001 |
| Access to cannabis | L vs. D * | 7.33 (1) | 0.007 |
|  | L vs. H * | 18.77 (1) | *p*<0.001 |
|  | D vs. H * | 38.50 (1) | *p*<0.001 |
| Friends disapprove: alcohol | L vs. D * | 24.47 (1) | *p*<0.001 |
|  | L vs. H * | 238.69 (1) | *p*<0.001 |
|  | D vs. H * | 288.79 (1) | *p*<0.001 |
| Friends disapprove: cannabis | L vs. D | 2.23 (1) | 0.14 |
|  | L vs. H * | 32.11 (1) | *p*<0.001 |
|  | D vs. H * | 43.23 (1) | *p*<0.001 |
| Moderate/high risk of harm due to use: alcohol | L vs. D * | 20.66 (1) | *p*<0.001 |
|  | L vs. H * | 4.28 (1) | *p*<0.001 |
|  | D vs. H * | 11.96 (1) | *p*<0.001 |
| Moderate/high risk of harm due to use: cannabis | L vs. D * | 22.68 (1) | *p*<0.001 |
|  | L vs. H * | 8.47 (1) | 0.004 |
|  | D vs. H * | 30.51 (1) | *p*<0.001 |

Notes: L (Low risk) = High friend disapproval and low perceived risk of harm; D (Divergent risk) = High friend disapproval and high perceived risk of harm; H (High risk) = Low friend disapproval and low perceived risk of harm; df= degrees of freedom;

* Statistically significant at *p<*0.05 after false discovery rate adjustment

Supplemental Table 3. Alcohol Expectancies model

Paired comparisons between latent class correlates:

| **Correlate** | **Paired class comparison** | **Wald (df)** | ***p*-value** |
| --- | --- | --- | --- |
| Age | L vs. D | 2.19 (1) | 0.14 |
|  | L vs. H | 0.23 (1) | 0.63 |
|  | D vs. H | 0.28 (1) | 0.60 |
| Black (vs Latino) | L vs. D | 0.02 (1) | 0.90 |
|  | L vs. H | 0.01 (1) | 0.92 |
|  | D vs. H | 0.00 (1) | 1.00 |
| Female (vs Male) | L vs. D | 4.51 (1) | 0.03 |
|  | L vs. H | 0.90 (1) | 0.34 |
|  | D vs. H | 0.28 (1) | 0.60 |
| Household income below $50K/year | L vs. D | 0.07 (1) | 0.79 |
|  | L vs. H | 1.01 (1) | 0.32 |
|  | D vs. H | 1.39 (1) | 0.24 |
| Household income >$100K/year | L vs. D | 3.90 (1) | 0.05 |
|  | L vs. H | 0.57 (1) | 0.45 |
|  | D vs. H | 0.32 (1) | 0.57 |
| ADI 2^nd^ quartile | L vs. D | 0.60 (1) | 0.44 |
|  | L vs. H | 0.58 (1) | 0.45 |
|  | D vs. H | 0.05 (1) | 0.82 |
| ADI 3^rd^ quartile | L vs. D | 0.69 (1) | 0.41 |
|  | L vs. H | 0.18 (1) | 0.67 |
|  | D vs. H | 1.04 (1) | 0.31 |
| ADI 4^th^ quartile | L vs. D | 0.57 (1) | 0.45 |
|  | L vs. H | 0.20 (1) | 0.65 |
|  | D vs. H | 0.94 (1) | 0.33 |
| Any alcohol through Follow-up 2 | L vs. D * | 13.98 (1) | *p*<0.001 |
|  | L vs. H | 2.26 (1) | 0.13 |
|  | D vs. H * | 18.38 (1) | *p*<0.001 |
| Importance of Religion | L vs. D | 0.98 (1) | 0.32 |
|  | L vs. H | 1.56 (1) | 0.21 |
|  | D vs. H | 3.52 (1) | 0.06 |

Supplemental Table 3, continued. Alcohol Expectancies model

| **Correlate** | **Paired class comparison** | **Wald (df)** | ***p*-value** |
| --- | --- | --- | --- |
| Internalizing | L vs. D | 4.12 (1) | 0.81 |
|  | L vs. H | 0.06 (1) | 0.83 |
|  | D vs. H | 1.53 (1) | 0.22 |
| Externalizing | L vs. D * | 15.14 (1) | *p*<0.001 |
|  | L vs. H | 0.86 (1) | 0.35 |
|  | D vs. H * | 12.94 (1) | *p*<0.001 |
| Peer prosocial | L vs. D | 0.19 (1) | 0.66 |
|  | L vs. H | 0.58 (1) | 0.45 |
|  | D vs. H | 0.18 (1) | 0.67 |
| Peer rule breaking | L vs. D | 1.86 (1) | 0.17 |
|  | L vs. H * | 17.28 (1) | *p*<0.001 |
|  | D vs. H * | 24.03 (1) | *p*<0.001 |
| Parental monitoring | L vs. D * | 7.36 (1) | 0.006 |
|  | L vs. H | 3.39(1) | 0.07 |
|  | D vs. H * | 14.05 (1) | *p*<0.001 |
| MACV FS | L vs. D | 0.23 (1) | 0.63 |
|  | L vs. H | 1.15 (1) | 0.28 |
|  | D vs. H | 1.84 (1) | 0.18 |
| MACV FO | L vs. D | 3.41 (1) | 0.06 |
|  | L vs. H | 3.79 (1) | 0.05 |
|  | D vs. H | 0.28 (1) | 0.59 |
| MACV FR | L vs. D * | 20.69 (1) | *p*<0.001 |
|  | L vs. H | 2.12 (1) | 0.15 |
|  | D vs. H | 2.57 (1) | 0.11 |
| **Outcome at 1-year** |  |  |  |
| Alcohol Positive Expectancies | L vs. D * | 15.04 (1) | *p*<0.001 |
|  | L vs. H | 0.43 (1) | 0.51 |
|  | D vs. H | 4.43 (1) | 0.03 |

Notes: L (Low risk) = High friend disapproval and low perceived risk of harm; D (Divergent risk) = High friend disapproval and high perceived risk of harm; H (High risk) = Low friend disapproval and low perceived risk of harm; df= degrees of freedom; $50K= $50,000; $100K=$100,000; MACV= Mexican American Cultural Values, FR= Family as referent, FO= Family obligations, FS= Family support

* Statistically significant at *p<*0.05 after false discovery rate adjustment

Supplemental Table 4. Cannabis Expectancies model

Paired comparisons between latent class correlates

| **Correlate** | **Paired class comparison** | **Wald (df)** | ***p*-value** |
| --- | --- | --- | --- |
| Age | L vs. D | 3.06 (1) | 0.08 |
|  | L vs. H | 0.25 (1) | 0.61 |
|  | D vs. H | 0.47 (1) | 0.49 |
| Black (vs Latino) | L vs. D | 0.26 (1) | 0.61 |
|  | L vs. H | 0.00 (1) | 0.96 |
|  | D vs. H | 0.09 (1) | 0.76 |
| Female (vs Male) | L vs. D | 3.65 (1) | 0.06 |
|  | L vs. H | 0.96 (1) | 0.33 |
|  | D vs. H | 0.11 (1) | 0.74 |
| Household income below $50K/year | L vs. D | 0.01 (1) | 0.90 |
|  | L vs. H | 0.74 (1) | 0.39 |
|  | D vs. H | 0.88 (1) | 0.35 |
| Household income >$100K/year | L vs. D | 4.23 (1) | 0.04 |
|  | L vs. H | 0.55 (1) | 0.46 |
|  | D vs. H | 0.40 (1) | 0.53 |
| Any cannabis through Follow-up 2 | L vs. D | 0.16 (1) | 0.69 |
|  | L vs. H | 4.03 (1) | 0.04 |
|  | D vs. H | 3.21 (1) | 0.07 |
| Internalizing | L vs. D | 3.54 (1) | 0.06 |
|  | L vs. H | 0.05 (1) | 0.83 |
|  | D vs. H | 1.28 (1) | 0.26 |
| Externalizing | L vs. D * | 17.09 (1) | *p*<0.001 |
|  | L vs. H | 0.94 (1) | 0.33 |
|  | D vs. H * | 14.34 (1) | *p*<0.001 |
| Peer prosocial | L vs. D | 0.17 (1) | 0.68 |
|  | L vs. H | 0.74 (1) | 0.39 |
|  | D vs. H | 0.30 (1) | 0.58 |
| Peer rule breaking | L vs. D | 1.89 (1) | 0.17 |
|  | L vs. H * | 15.07 (1) | *p*<0.001 |
|  | D vs. H * | 21.97 (1) | *p*<0.001 |

Supplemental Table 4, continued. Cannabis Expectancies model

| **Correlate** | **Paired class comparison** | **Wald (df)** | ***p*-value** |
| --- | --- | --- | --- |
| Parental monitoring | L vs. D | 7.98 (1) | .005 |
|  | L vs. H | 3.58 (1) | 0.06 |
|  | D vs. H * | 15.16 (1) | *p*<0.001 |
| MACV FS | L vs. D | 0.20 (1) | 0.66 |
|  | L vs. H | 0.99 (1) | 0.32 |
|  | D vs. H | 1.59 (1) | 0.21 |
| MACV FO | L vs. D | 3.52 (1) | 0.06 |
|  | L vs. H | 4.50 (1) | 0.03 |
|  | D vs. H | 0.48 (1) | 0.49 |
| MACV FR | L vs. D * | 21.88 (1) | *p*<0.001 |
|  | L vs. H | 2.59 (1) | 0.11 |
|  | D vs. H | 2.33 (1) | 0.13 |
| **Outcome at 1-year** |  |  |  |
| Cannabis Positive Expectancies | L vs. D * | 28.53 (1) | *p*<0.001 |
|  | L vs. H | 0.66 (1) | 0.42 |
|  | D vs. H * | 8.08 (1) | .004 |

Notes: L (Low risk) = High friend disapproval and low perceived risk of harm; D (Divergent risk) = High friend disapproval and high perceived risk of harm; H (High risk) = Low friend disapproval and low perceived risk of harm; df= degrees of freedom; $50K= $50,000; $100K=$100,000; MACV= Mexican American Cultural Values, FR= Family as referent, FO= Family obligations, FS= Family support

* Statistically significant at *p<*0.05 after false discovery rate adjustment

Supplemental Analyses: Excluding youth who endorsed lifetime use of alcohol or cannabis

Since prior experiences with alcohol and cannabis use could have an impact on alcohol and cannabis expectancies, we examined results when excluding participants who endorsed lifetime use of alcohol or cannabis.

To address this issue, we first examined the latent classes in which youth were most likely to be classified in the selected 3-class model if they reported lifetime alcohol (n=699) or cannabis use (n=14). As might be expected, youth who reported any alcohol (including a sip or taste) by Follow-up 2 were more likely to be classified in the “high risk” class (30.2%), with all latent classes differing from each other in expected directions in the proportion of youth endorsing lifetime alcohol use (“low risk”: 23.2%; “divergent” precursor endorsement: 16.0%; p<.05). Youth who reported any lifetime cannabis use by Follow-up 2 (n=14) were more likely to be in the “high risk” (vs “low risk” or “divergent” classes (p<.05). Only 3 of the 14 youth who reported lifetime cannabis use by Follow-up 2 did not report any lifetime alcohol use by Follow-up 2. Thus, while youth who reported lifetime alcohol or cannabis use were distributed across all 3 classes, as expected, more youth who reported any lifetime alcohol or cannabis use were classified in the “high risk” (vs “low risk” and “divergent” risk classes).

Analyses that excluded youth who reported lifetime alcohol or cannabis use by Follow-up 2 (N=2,629) indicated selection of a 3- or 4-class model based on consideration of multiple fit indices, including avoiding classes with a small proportion of cases (e.g., <5%) (Nylund-Gibson & Choi, 2018). At 5 or more classes, latent classes included <5% of cases. The 3-class model identified classes which were very similar to the 3 classes identified when including all youth (i.e., youth who did and did not report lifetime alcohol or cannabis use), see figures below, supporting the original analysis using all cases. Notably, there was slightly higher average posterior probability of assignment to the most likely class for the 3- (vs 4-class) model, supporting selection of the 3-class (vs 4-class) model. Specifically, in the 3-class model, average posterior probability of assignment to the most likely class was for class 1: 86.17 (SE=.002), class 2: 88.74 (SE=.003), and class 3: 88.23 (SE=.013). For the 4-class model, average posterior probability of assignment to the most likely class was for class 1: 82.86 (SE=.005), class 2: 84.18 (SE=.005), class 3: 82.95 (SE=.016), and class 4: 75.20 (SE=.020). In sum, these results, excluding youth who reported lifetime alcohol or cannabis use, resulted in a similar 3-class model to that when including all cases (i.e., with and without lifetime alcohol and cannabis use).

In addition, cross-classification of cases in the 3- and 4-class models (analyses that excluded youth who reported lifetime alcohol or cannabis use) indicated that no “high risk” cases in the 3-class model were classified as “low risk” in the 4-class model. Further, most (99%; 1096 out of 1110) of the “low risk” cases in the 3-class model continued to be classified in the “low risk” class in the 4-class model, with a very small number (n=14) being classified in the “new” 4^th^ class. Taken together, these results suggest that a 3-class model also provides a parsimonious, adequate representation of youth who reported no lifetime use of alcohol and cannabis by Follow-up 2.

| **Classes** | **LL** | **BIC(LL)** | **AIC(LL)** | **N par** | **L²** | **df** | **p-value** | **Class. Err.** | **Entropy R²** |
| --- | --- | --- | --- | --- | --- | --- | --- | --- | --- |
| 1 | -6304.10 | 12655.448 | 12620.20 | 6 | 1187.011 | 90 | p<.00001 | 0 | 1 |
| 2 | -5985.46 | 12073.279 | 11996.91 | 13 | 549.7211 | 83 | p<.00001 | 0.14 | 0.51 |
| 3 | -5833.44 | 11824.374 | 11706.89 | 20 | 245.6948 | 76 | p<.00001 | 0.12 | 0.63 |
| 4 | -5769.98 | **11752.577** | 11593.97 | 27 | 118.7779 | 69 | p<.00018 | 0.17 | 0.61 |
| 5 | -5745.59 | 11758.909 | 11559.18 | 34 | 69.9893 | 62 | 0.23 | 0.14 | 0.65 |
| 6 | -5735.37 | 11793.593 | **11552.74** | 41 | 49.5527 | 55 | 0.68 | 0.16 | 0.62 |
| 7 | -5731.23 | 11840.437 | 11558.47 | 48 | 41.2764 | 48 | 0.75 | 0.19 | 0.57 |
| 8 | -5727.45 | 11887.984 | 11564.90 | 55 | 33.7031 | 41 | 0.78 | 0.20 | 0.58 |
| 9 | -5725.66 | 11939.532 | 11575.32 | 62 | 30.1301 | 34 | 0.66 | 0.26 | 0.51 |

| **Classes** | **LRT (df=7)** | **LRT**  **p-value** | **VLRT** | **VLRT**  **p-value** | **Bootstrap p-value** |
| --- | --- | --- | --- | --- | --- |
| 1 |  |  |  |  | <.01 |
| 2 | 637.29 | <.01 | 637.29 | 0 | <.01 |
| 3 | 304.03 | <.01 | 304.03 | 0 | <.01 |
| 4 | 126.92 | <.01 | 126.92 | 0 | <.01 |
| 5 | 48.79 | <.01 | 48.79 | 0 | <.01 |
| 6 | 20.44 | <.01 | 20.44 | 0.04 | <.01 |
| 7 | 8.28 | >.05 (ns) | 8.28 | 0.13 | 0.02 |
| 8 | 7.57 | >.05 (ns) | 7.57 | 0.15 | 0.02 |
| 9 | 3.57 | >.05 (ns) | 3.57 | 0.46 | 0.01 |


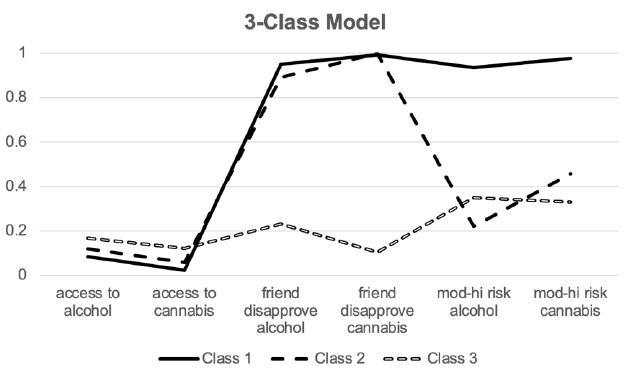

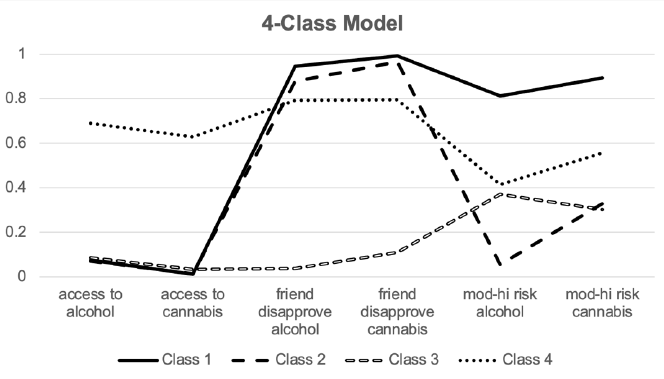


Technical note:

Regarding detection of significant pairwise effects within an outcome (e.g., high vs low risk classes in predicting positive alcohol expectancies), and across outcomes (e.g., high risk vs divergent class in predicting positive alcohol vs cannabis expectancies): In brief, class-specific standard errors (SE) help explain imprecision of pairwise contrasts (e.g., when looking within an outcome), while the SE of the between-group contrast determines whether between-group differences are statistically significant (Gudicha et al., 2017), which is useful to consider when looking across outcomes. We also note that in this study’s 3-step analyses using nested and weighted data, effective statistical power for pairwise contrasts is influenced not only by the count of cases in a class, but by multiple factors, such as design effects (e.g., nesting of cases in families and site), class imbalance (i.e., uneven group size), and classification uncertainty (Gudicha et al., 2017; Dziak et al., 2016; Bakk & Vermunt, 2016).

In this study’s analyses, the high risk class was the smallest of the 3 latent classes (which included two larger classes: low risk and divergent), resulting in imbalanced classes (uneven group sizes). Due to class imbalance and factors associated with being the smallest class, pairwise comparisons involving the high risk class generally had less precision and lower statistical power relative to the two larger classes (Gudicha et al., 2017). In this context, likely reasons for the absence of a significant difference between the low and high risk classes for the alcohol expectancies outcome are (1) the relatively small size of the high risk class (i.e., class imbalance) and (2) the high risk class’s relatively large SE (0.07; double that of the low risk class: SE=0.03). The high risk class’s larger SE indicates a relatively imprecise estimate for the high (vs low) risk class in predicting the alcohol expectancies outcome. The high risk class’s larger SE suggests that estimates for this smaller class are unstable, have wider confidence intervals, and that pairwise comparisons involving this class are sensitive to small changes due to wide confidence intervals (Gudicha et al., 2017).

Specifically, for the positive alcohol expectancies outcome, comparison of the two larger classes (low and divergent risk) resulted in a strong effect (Wald [df=1]=15.04, p<.001), whereas comparison involving the smallest class (e.g., divergent vs high risk) was nominally significant (Wald [df=1] = 4.43, p=.035) and did not survive correction for multiple testing. The true difference in alcohol expectancies between the divergent and high risk classes was likely small and challenging to detect with adequate precision given the size of the relatively small high risk class and the influence of other factors (e.g., nested sampling structure, probabilistic assignment of cases to classes) in the 3-step model.

When looking across outcomes (positive alcohol and cannabis expectancies), we note that statistical significance depends not only on the observed difference in class-specific means, but on the effective standard error (SE) of the difference between groups (not just the class-specific SEs). The SE for the difference between groups (i.e., the pairwise contrast) accounts for variability in class estimates and their covariance. Notably, the SE of the pairwise contrast is influenced by class imbalance and sample weighting, as well as classification uncertainty (i.e., probabilistic assignment to a latent class), which can additionally widen the SE of pairwise contrasts (Gudicha et al., 2017).

For the cannabis positive expectancies outcome, a difference in the pairwise comparison between the high risk and divergent classes was found to be statistically significant (Wald [df=1] = 8.08, p<.001), whereas the pairwise comparison for the two classes in predicting alcohol expectancies was nominally significant (Wald [df=1] = 4.43, p=.035), but did not survive correction for multiple testing. For both alcohol and cannabis expectancies outcomes, mean differences between high risk and divergent classes were small (alcohol: 0.08 vs 0.07, respectively, mean difference = 0.01; cannabis: 0.11 vs 0.10, respectively, mean difference = 0.01), and class-specific standard errors (SE) were larger for the high risk (vs divergent) class for both outcomes (alcohol: 0.06 vs 0.03, respectively; cannabis: 0.07 vs 0.03, respectively). However, statistical significance for the pairwise comparison depends on the SE of the pairwise contrast, rather than class-specific estimates of SE (Gudicha et al., 2017). As noted above, the SE of the pairwise contrast is influenced by multiple factors (e.g., combined variance and covariance of the class-specific estimates which differ across outcomes, classification uncertainty, class imbalance) in the 3-step model. The SE of the difference for the pairwise contrast between high risk and divergent groups for the cannabis (vs alcohol) positive expectancy outcome was smaller (.0035 vs .0048, respectively), which provided greater precision to detect a between-group difference (high risk vs divergent classes) for the cannabis (vs alcohol) outcome (Gudicha et al., 2017).

References

Gudicha DW, Schmittmann VD, Vermunt JK. Statistical power of likelihood ratio and Wald tests in latent class models with covariates. Behav Res Methods. 2017 Oct;49(5):1824-1837.

Vermunt, J.K. (2010). Latent class modeling with covariates: Two improved three-step approaches. Political Analysis, 18, 450-469.

Dziak JJ, Bray BC, Zhang J, Zhang M, Lanza ST. Comparing the Performance of Improved Classify-Analyze Approaches For Distal Outcomes in Latent Profile Analysis. Methodology (Gott). 2016 Oct;12(4):107-116.

Bakk, Z., & Vermunt, J. K. (2016). Robustness of stepwise latent class modeling with continuous distal outcomes. Structural Equation Modeling, 23(1), 20–31.
